# A Multilayer Paste Based on Ag Nanoparticles with Cu@Sn for Die Attachment in Power Device Packaging

**DOI:** 10.3390/ma15030914

**Published:** 2022-01-25

**Authors:** Jintao Wang, Xinjie Wang, Lin Zhang, Luobin Zhang, Fangcheng Duan, Fengyi Wang, Weiwei Zhang, Jianqiang Wang, Zheng Zhang, Chunjin Hang, Hongtao Chen

**Affiliations:** 1 Department of Materials Science and Engineering, Harbin Institute of Technology (Shenzhen), Shenzhen 518055, China; jintaowcqu@foxmail.com (J.W.); XinJieW@hit.edu.stu.cn (X.W.); LinZhang@hit.edu.stu.cn (L.Z.); LuobinZ@hit.edu.stu.cn (L.Z.); DFCheng@hit.edu.stu.cn (F.D.); WangFW@hit.edu.stu.cn (F.W.); WeiWeiZ@hit.edu.stu.cn (W.Z.); JQWang@hit.edu.stu.cn (J.W.); ZhengZhang@hit.edu.stu.cn (Z.Z.); 2 State Key Lab of Advanced Brazing and Joining, Harbin Institute of Technology, Harbin 150001, China; hangcj@hit.edu.cn; 3 Sauvage Laboratory for Smart Materials, Harbin Institute of Technology (Shenzhen), Shenzhen 518055, China

**Keywords:** TLP, Cu@Sn, integrated packaging, soldering

## Abstract

A 3–5 μm Cu@Sn core-shell powder was prepared by chemical plating. Based on the mixture of this Cu@Sn and Ag NPs (nanoparticles), a soldering material for third-generation semiconductors was prepared. The joints prepared with this soldering material had a shear strength of over 40 MPa at 25 °C. This joint did not fail after more than 600 thermal cycles from −40 °C to 140 °C. The special feature of this joint is that the energy potential difference between nanoparticles and micron particles generated in the surface force field during reflow promoted the surface pre-melting of the particles by releasing the excess energy. By this mechanism, it was possible to reduce the porosity of the sintered layer. At the same time, due to the high surface activity energy of nano-silver, the diffusion of the Sn atoms was promoted, further enhancing the bond strength.

## 1. Introduction

The third generation of wide band semiconductor materials represented by silicon carbide (SiC) and gallium nitride (GaN) has the advantages of high breakdown voltage, high power density, chemical resistance, high electron mobility and small dielectric constant, which enable SiC chips to achieve higher computing efficiency and withstand harsher service environments [1]. The widely used thermal interface material for SiC devices in practical applications is a gold-based soldering alloy. The most mainstream soldering alloy is Au80Sn, with a eutectic melting point of 280 °C [2]. High costs constrain the rapid development of SiC devices [3,4,5].

Low-temperature sintered silver nanoparticle technology is currently the most promising alternative to traditional soldering materials, other than Ag80Sn alloy soldering materials, for the application of thermal interface materials in wide-band semiconductor device packaging. It has a much greater potential for application because its performance is much better than that of gold–tin alloy soldering materials. Silver nanoparticles refer to silver metal particles with a particle size below 100 nm. When the size of silver particles reaches the nanometer level, the nanoparticles have a considerable surface energy, which makes the silver nanoparticles sinter together by releasing surface energy at a temperature far below the melting point of the metal. When the silver nanoparticles are of the right size, the sintering temperature can be reduced to about 200 °C [6,7,8].

The application of multi-scale silver pastes has received increasing attention in recent years. This micro–nano mixture undergoes plastic deformation and is less susceptible to fracture, just as fiber-reinforced composites. The addition of silver nanoparticles to silver nanoparticle solder paste can produce a micro–nano mixture such as that of fiber-reinforced composites, through which plastic deformation can occur and cracking can be inhibited. A silver composite paste with micron-sized silver flakes as the skeleton and silver nanoparticles as the binder has been proposed to change the sintering process from being surface diffusion-driven to lattice diffusion-driven to improve the sintering drive [2,6,9]. In addition, Tian et al. mixed Ag NPs with sizes of 30–50 nm and submicron Ag particles to tightly stack Ag NPs in paste [10].

In general, the reduction in shear strength of sintered Ag NP joints during high-temperature service or thermal cycling is caused by the shrinkage of the sintered microstructure. The reduction in strength is caused by the overall shrinkage of the joint, resulting in secondary sintering. Both microstructural shrinkage and macroscopic structural shrinkage result in the generation of cracks. As a thermal interface material for chip packaging, it becomes very critical to solve the shrinkage problem of silver nanoparticle sintered joints during high-temperature service. Therefore, how to solve the shrinkage problem of silver nanoparticle sintered joints during high-temperature service becomes a very critical issue [11,12,13].

In summary, at present, the most promising nano-silver sintering method is micron silver–nano-silver hybrid sintering, but this method is limited by the cost. So, aiming to replace micron silver particles, we developed a Cu@Sn micron particle.

It is worth noting that Cu@Sn has a significant price advantage over micron Ag particles of the same size. At a lower cost, Cu@Sn can not only perform the same role as micron silver particles in making a tighter stack of Ag NPs in the paste, but it can also perform its unique role. In Cu@Sn, the outer Sn layer of the particles is conducive to the densification of the sintered layer and enhances the shear strength and conductivity of the sintered layer; at the same time, Sn reduces the initial sintering temperature and accelerates the growth of sintering neck. In addition, the electromigration time of sintered Ag–Sn bimetallic nanoparticles is more than ten times that of sintered Ag NPs. The combination of TLP technology and nano-silver sintering technology undoubtedly allows the nano-silver sintering technology to have a broader development direction.

## 2. Experimental Materials and Method

The chemicals used in the experiment are shown in Table 1.

### 2.1. Cu@Sn and Ag NPs Preparation

The sodium citrate solution and the ferrous sulfate solution were evenly mixed to make a reducing solution, which was added drop by drop to the silver nitrate solution with strong mechanical stirring. After the reaction was completed, the reduced silver nanoparticles were separated from the solution by centrifugal separation.

Thiourea, ascorbic acid, sodium hypophosphite, hydroquinone, polyethylene glycol and disodium EDTA were added to deionized water (180 mL). The weight ratio of these substances was 60:10:30:1:1:1. Then, stannous chloride was added dropwise to the solution and concentrated hydrochloric acid was added dropwise to adjust the pH of the plating solution to 1.0. The mass ratio of stannous chloride to ascorbic acid was 1:1. The cleaned Cu powder was poured into the mixed plating solution with a stirring rate of 200 rpm for 1 h. The obtained mixture was placed in a funnel and filtered and the obtained metal powder was washed with deionized water and anhydrous ethanol three times each; then, we obtained Cu@Sn core-shell structure metal powder after vacuum drying.

The principle of chemical plating is shown in the equation below. The mechanism is to add thiourea to the chemical plating solution to lower the electrode potential of Cu^2+^/Cu to below the electrode potential of Sn^2+^/Sn. The continuous precipitation of Sn can be realized under the action of reducing agent afterwards, achieving the purpose of plating Sn on Cu powder [14,15,16].

Sn^2+^ + H_2_O ⇆ Sn (OH)^+^ ↓ + H^+^

Sn^2+^ + Cu + 2(CH_4_N_2_S) = Sn + [Cu (CH_4_N_2_S)_2_]^2+^

### 2.2. Soldering

**Figure 1:**
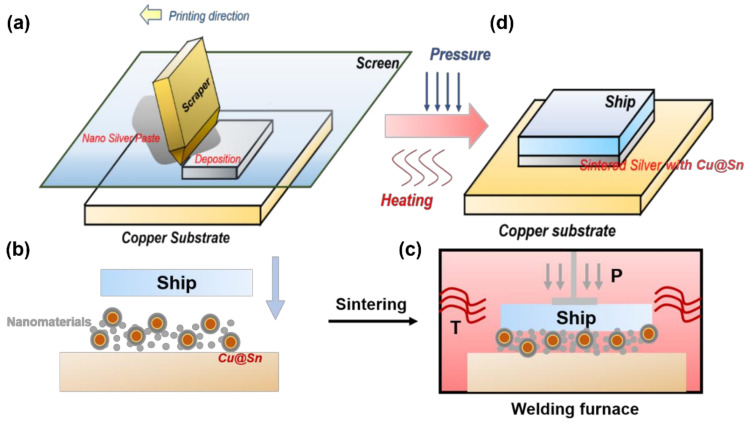
Schematic diagram of reflux process. (**a**) Screen printing diagram. (**b**) Paste on the substrate after screen printing. (**c**) Reflux schematic diagram. (**d**) Diagram of the obtained joint.

The composite slurry was prepared as follows: Firstly, the prepared alcohol-based silver nanoparticle was mixed with pine oil alcohol in a 5:1 mass ratio under ultrasonic stirring. Then, the Cu@Sn nanoparticles and pine oil alcohol were mixed thoroughly under the action of ultrasonic stirring at a mass ratio of 7:1. The two well-mixed slurries were mixed and continued with sufficient ultrasonic stirring. Subsequently, to the mixed slurry containing silver nanoparticles and Cu@Sn particles, additives such as Span85 and salicylic acid were added and mixed thoroughly under the action of ultrasonic stirring. The paste was applied to the copper substrate using stencil printing and refluxed at 250 °C for 10 min using a 5 MPa auxiliary pressure (Figure 1). Meanwhile, the joints of control groups refluxed at 0 MPa and 10 MPa.

### 2.3. Testing

The morphology of Ag NPs and Cu@Sn particles was observed by transmission electron microscopy (TEM; TecnaiG2F30, Philips-FEI, Groningen, Netherlands). The microstructure of the particles, solder joints and shear fracture surfaces was characterized using a focused ion beam/scanning electron microscope (FIB/SEM; HELIOS 600i, Philips-FEI, Groningen, Netherlands) equipped with an electron dispersive X-ray detector (EDX; XM4, Philips-FEI, Groningen, The Netherlands). The composition of the Cu@Sn particles before and after sintering was characterized by X-ray diffractometry (XRD; D/max 2500; Rigaku, Tokyo, Japan). The melting points of the different phases in the solder joints were measured with a differential scanning calorimeter (DSC; STA 449F5, NETZSCH, Selb, Germany) at a heating rate of 10 °C·s^−1^. To verify the long-term service reliability of the solder joints at high temperatures, the samples were subjected to aging tests at 300 °C using a muffle furnace and the weld microstructure and mechanical properties of the samples were examined at 300, 600 and 900 h, respectively. A creep tester (SANS; GWTA-105, Suzhou, China; 100 kg) was used to measure the shear strength of the welded joints at room temperature at a shear rate of 0.25 mm·s^−1^. The sheared sample was a 5 × 5 × 2 (mm) copper substrate soldered to a 10 × 10 × 2 (mm) copper substrate. A high-temperature creep tester was used to measure the shear strength of solder joints at 400 °C.

## 3. Experimental Results and Discussion

### 3.1. Cu@Sn Particles and Ag NPs of 3.0–5.0 μm

In the study, sodium citrate dihydrate was used as the reducing agent and silver nitrate as the silver source and the reaction yielded silver nanoparticles of pure composition. The hydroxyl group was oxidized by Ag^+^ during the reaction to obtain NaOOCCH_2_COCH_2_COONa and CO_2_, and Ag^+^ was reduced to Ag atoms [7,8,17]. The reaction process is shown below.

Ag^+^ + Na_3_C_6_H_5_O_7_2H_2_O → Ag + Na_2_C_5_H_4_O_5_ + CO_2_ + H^+^

**Figure 2:**
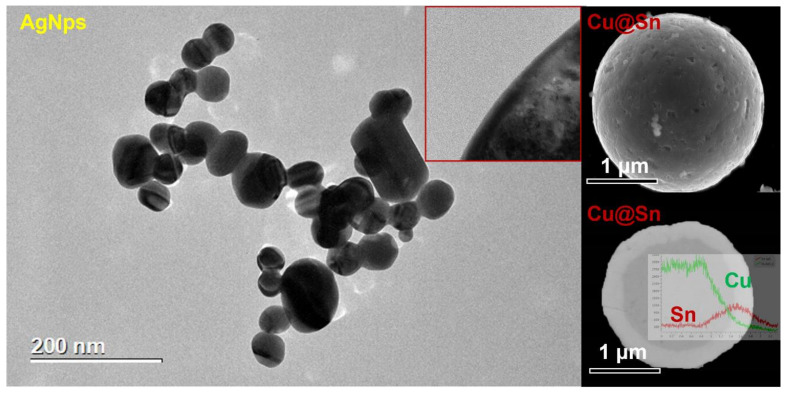
TEM images of Ag NPs and SEM images of Cu@Sn particles.

Sodium citrate and silver nitrate were uniformly dispersed in the reaction solution and the silver nanoparticles with controlled morphological size were prepared by controlling the ratio of reactants and reaction conditions. During the reaction, part of the sodium citrate reduced Ag^+^ to monomeric Ag and the remaining sodium citrate gradually adsorbed on the surface of the silver atoms, preventing the silver particles from continuing to grow. The silver particles grew to the nanometer size and were completely coated by sodium citrate, stopping the growth of silver particles from reaching the nanometer size (Figure 2) [18].

**Figure 3:**
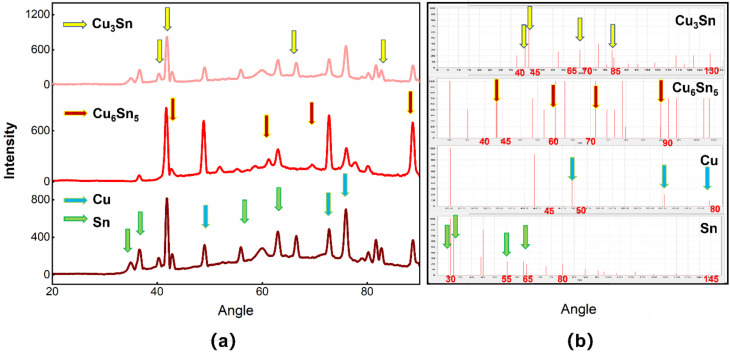
(**a**) XRD patterns of Cu@Sn particles. The XRD patterns were derived from multiple replicate experiments. (**b**) JCPDS cards of Cu_3_Sn, Cu_6_Sn_5_, Cu and Sn.

The Cu@Sn particles were obtained through three stages of reduction, Cu deposition of Cu–Sn and autocatalytic deposition of Sn. Figure 2 shows the morphology of the Cu particles after having chemically plated Sn. After chemical plating, the scallop-shaped Sn shell covered the surface of the smooth Cu core. As shown in Figure 2, an EDX scan analysis was performed at the Cu–Sn interface and the Cu content was detected, at the beginning of the scan line, to be close to 100% and to have decreased to zero at the end of the scan line. The average diameter of the Cu particles was 4 μm and the thickness of the Sn shell was about 1 μm. Cu_6_Sn_5_ formed between the interfacial layers of Cu and Sn at a thickness of about eighty nanometers. Moreover, the Cu_3_Sn between Cu nuclei and Cu_6_Sn_5_ had a thickness of tens nanometers. In addition, the XRD diffraction data of the Cu@Sn particles were analyzed (Figure 3) and the Cu_6_Sn_5_ and Cu_3_Sn IMC (intermetallic compound) appeared in the core-shell structure.

### 3.2. Joints

**Figure 4:**
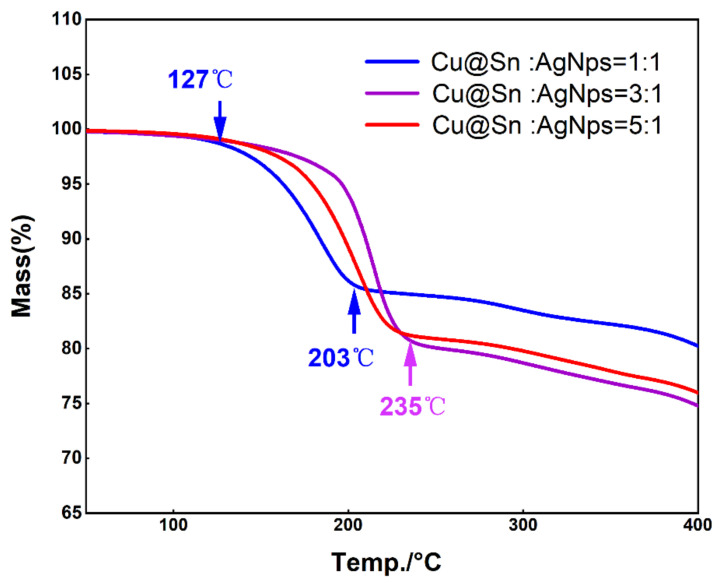
TGA curves of different pastes.

The complete removal of the organic dispersant led to a porous microstructure. In the early stages of the sintering process, these interconnected voids maintained their nanoscale dimensions until this point, when the main sintering mechanism was surface diffusion. It is noteworthy that, according to the TGA (thermogravimetric analysis) curves in Figure 4, the organic shell started to decompose at 127 °C. According to the TGA curve, this process occurred from 127 °C to 235 °C (Figure 4). Moreover, while most of the particles retained their initial morphology, the integration of some small and large particles was visible. Above 200 °C, the openings became isolated, and the silver particles were further sintered together by Ostwald ripening, leading to the formation of larger irregular particles and to the densification of the silver layer. More silver nanoparticles meant lower activation energy, that is, the high surface energy of the silver nanoparticles allowed the soldering material to be sintered with less activation energy.

**Figure 5:**
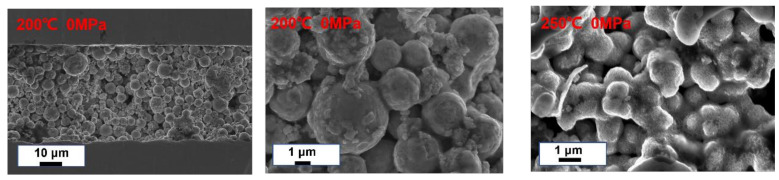
Joints obtained by refluxing at 0 MPa auxiliary pressure (200 °C and 250 °C). No dense microstructure was formed, which illustrates the need for auxiliary pressure.

During solvent evaporation, the solid content of silver nanoparticles increased and closer contact among silver nanoparticles occurred, which facilitated the occurrence of sintering. Adjacent particles started to bond freely and interconnect (hard agglomerates) to form chains. However, for most particles, no significant change in particle size/shape was observed compared to the pristine silver particles in the slurry, as there was not enough driving force to form dense silver joints in the absence of pressure (Figure 5).

**Figure 6:**
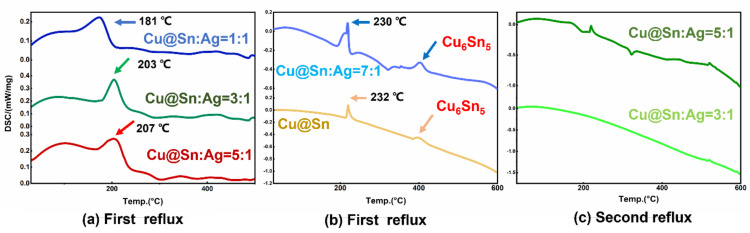
DSC curves of different pastes. DSC experiments showed that the Ag NPs in the paste allowed Sn to melt at temperatures below the melting point (231 °C).(**a**) First reflux, (**b**) First reflux, (**c**) Second reflux.

At 203 °C, the DSC curve demonstrated a melting peak where the high surface energy of the silver nanoparticles allowed the Sn to melt at temperatures below the melting point (Figure 6). As the mass ratio of the Cu@Sn to Ag NPs increased, the reaction temperature gradually rose until it approached the melting point of Sn. In both secondary reflows, the reaction peaks no longer appeared, indicating that the joint could be reflowed at low temperatures and served at high temperatures using this soldering material.

After 10 min of reflux at 250 °C and 5 MPa auxiliary pressure, an apparently dense joint formed. The Sn atoms were diffused to almost the entire joint and the Cu nuclei became the “skeleton”, which the silver nanoparticles filled to enhance the bonding among the Cu@Sn particles.

**Figure 7:**
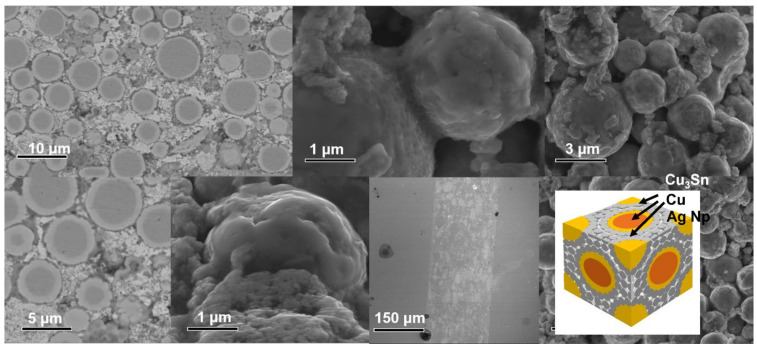
SEM images of joints (Cu@Sn: Ag NPs = 3:1, wt.%).

**Figure 8:**
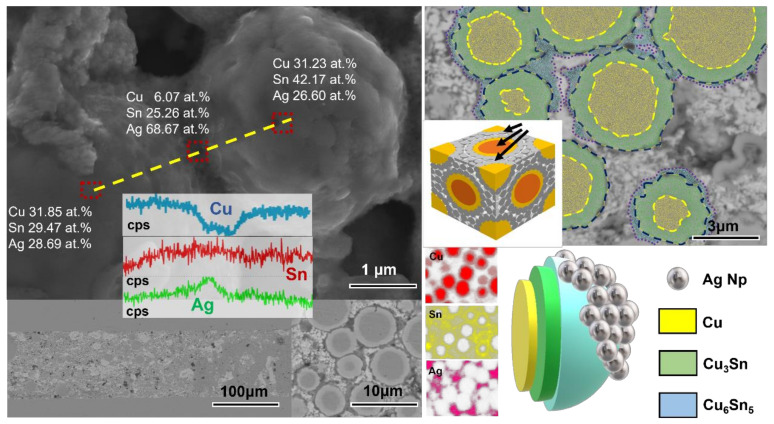
EDX images of joints; joint reflux at 250 °C for 10 min using 5 MPa auxiliary pressure. (Cu@Sn: Ag NPs = 3:1, wt.%).

The high interfacial energy of the porous sintered silver structures could be attributed to the large number of microdefects such as grain boundaries and dislocations, which provided many short-range diffusion channels and accelerated the diffusion of Sn ions. In addition, these surface and intergranular silver atoms were highly reactive. The chemical bonding between activated silver and tin was promoted, which is thought to be the result of the presence of nanoparticles (Figure 7 and Figure 8).

Here, we observed neck formation on the particle boundary in the thickness direction, which could be attributed to finer nanoparticles. The energy potential difference between the Ag NPs and the micron Cu@Sn particles generated in the surface force field promoted the surface pre-melting of the particles by releasing excess energy. At the same time, some of the original particles grew into larger particles with internal voids through the rearrangement of particles and clusters of nanoparticles. Through this mechanism, the porosity of the sintered layer may be reduced [19,20]. The reduced porosity in the sintered layer results in fewer sources of cracking and higher service reliability of the joint.

Both neck coarsening and pore closure increased significantly. This occurs when lattice and grain boundary diffusion dominate the sintering process, allowing significant mass transfer and diffusion. Due to the increased diffusion coefficient, the two different particle size groups eventually fused into a nearly solid structure. Driven by the high surface energy of the nanoparticles, the small particles diffused through the lattice and grain boundaries to form incorporated particles, which also acted as a bridge enabling the formation of necks between the particles. Finally, the rearrangement process of these particles facilitated the reduction in porosity and dense sintered structures.

However, the presence of micro-Cu@Sn reduced the sintering drive of the nano-silver paste and required higher processing temperatures to obtain reliable sintered joints.

### 3.3. Thermal Cycling

**Figure 9:**
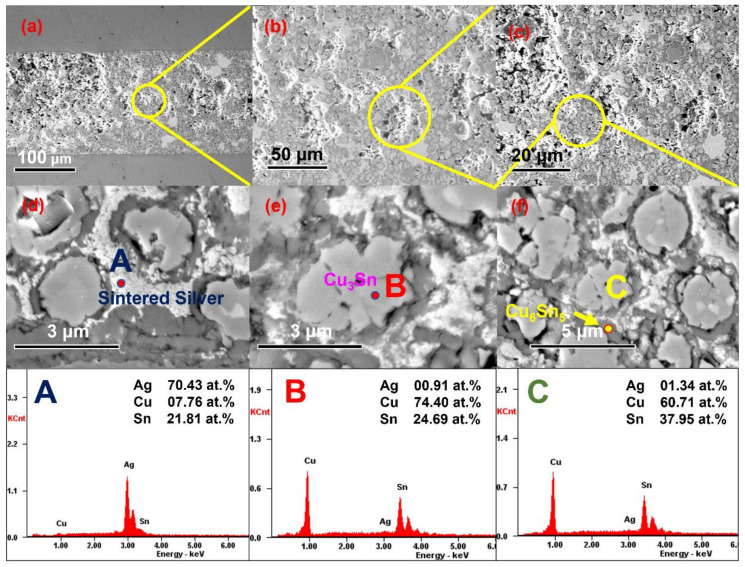
SEM images of the joint after 600 thermal cycles (Cu@Sn: Ag NPs = 3:1, wt.%). (**a**) Overall SEM image of the joint. (**b**) Partial zoom image. (**c**) Sintered nano-silver buffered the thermal stress from CTE mismatch. (**d**) EDX image of sintered silver. (**e**) EDX image of Cu_3_Sn (point scan). (**f**) EDX image of Cu_6_Sn_5_; A,B,C represent the point scans of the three points A,B,C.

Figure.9 shows the microstructure of the joints before and after 600 thermal shock cycles (−40–140 °C). All joints initially had nanoscale porous structures prior to thermal shock testing. These unique structures can release the thermo-mechanical stresses caused by the coefficient of thermal expansion (CTE) mismatch between the chip and the substrate during thermal cycling. The diffusion of Sn atoms allowed the joint to be almost completely IMCed during thermal cycling and the sintered neck between the sintered silver particles was filled with Sn atoms. This had the effect of buffering the stresses caused by CTE.

During the coarsening phase, the sintered Ag tended to aggregate and form micron-sized sintered bodies, as shown in Figure 9. The subsequent volume shrinkage led to the creation of voids and cracks. The thermomechanical stresses caused by the CTE mismatch could accelerate the propagation of voids and cracks and even lead to interfacial debonding. During the aging tests, the Cu@Sn particles embedded in the sintered joints acted as “pegs” to hold the sintered Ag aggregates together and prevent overgrowth. This limited the coarsening of the sintered silver to an acceptable level even under harsh conditions and prevented voids and cracks.

### 3.4. Shear Strength

**Figure 10:**
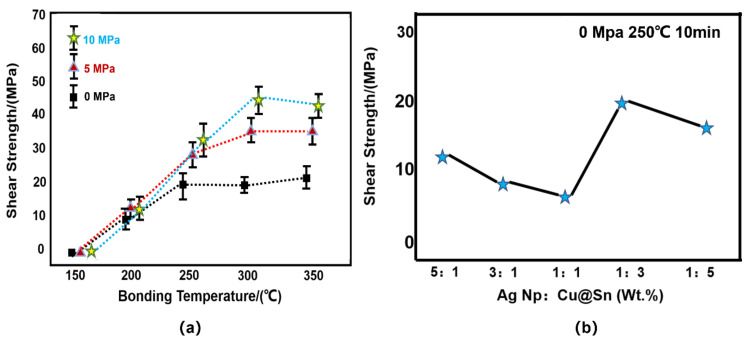
(**a**) Joint shear strength after reflowing of different pressures and different temperatures. (**b**) Strength of joints with different mass ratios of Cu@Sn to Ag NPs after reflowing at 0 MPa.

Shear strength tests demonstrated the outstanding strength and reliability of these joints (Figure 10). The shear strength of the joint increased with the increase in reflow temperature. In addition, an increase in auxiliary pressure during reflow increased the shear strength of the joint. A joint reflowed at 250 °C at a 5 MPa auxiliary pressure for 10 min could achieve a shear strength of 28.7 MPa.

**Figure 11:**
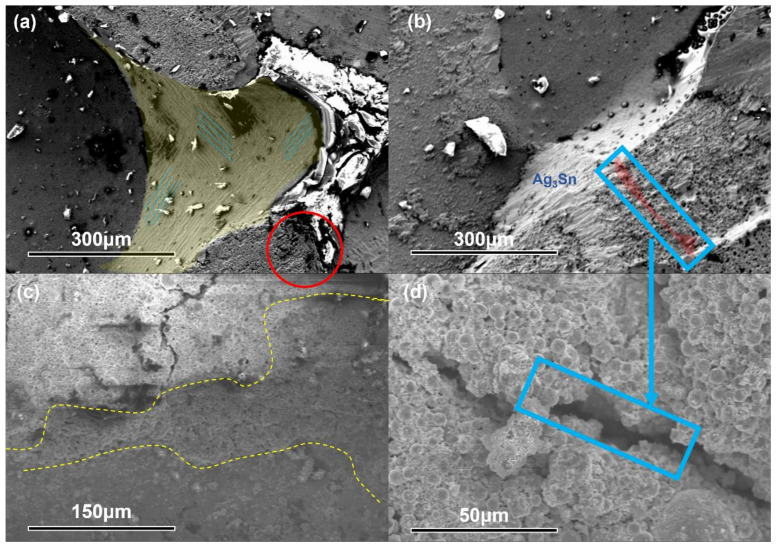
Fracture SEM images (Cu@Sn: Ag NPs = 3:1, wt.%); reflux at 250 °C, 5 MPa. (**a**) Smooth and undistorted fractures. (**b**) Micro-voids formed during the sintering stage were elongated. (**c**) Step-like fracture surface. (**d**) Voids expanded to become cracks.

The fracture surface of the joint was observed to study the fracture pattern. As seen in Figure 11, the morphology of the grains can be distinguished and the fracture was smooth with no significant deformation. The plastic deformation of the fracture indicates that the joint could withstand strong mechanical shocks, which is consistent with a high shear strength. When loaded with shear strength, the micro-voids formed during the sintering stage were elongated and necking occurred at the sintered neck between the voids. As the shear strength increased, the necking area failed and a crater-like fracture morphology was formed. When Cu@Sn particles were added, the fracture mode of the joint changed to brittle fracture. Although the Ag + Cu@Sn joints showed a cross-sectional microstructure similar to that of the sintered Ag joints, the decrease in shear strength was mainly attributed to defects caused by the addition of Cu@Sn particles.

## 4. Conclusions

A new high-temperature lead-free soldering material is proposed. It was made by mixing 3–5 μm Cu@Sn particles with Ag NPs. The Cu@Sn particles were prepared by a new chemical coating method and the organic solvent for mixing Cu@Sn with Ag NPs is also described. This soldering material was refluxed at 250 °C for 10 min at a 10 MPa pressure to obtain joints with a shear strength greater than 40 MPa. The microstructure within the joint was mainly Cu_3_Sn and Ag_3_Sn IMCs.

During reflow, the energy potential difference between nanoparticles and micron particles generated in the surface force field promoted surface pre-melting of the particles by releasing excess energy. At the same time, some of the original particles grew into large particles with internal voids through particle rearrangement and clustering of nanoparticles. By this mechanism, it was possible to reduce the porosity of the sintered layer.

## Figures and Tables

**Table 1:** Chemicals for experiments.

No.	Chemicals	Formula	Manufacturers
1	Disodium EDTA (AR)	C_10_H_14_N_2_Na_2_O_8_	Aladdin, China
2	Thiourea (AR)	CH_4_N_2_S	Aladdin, China
3	Sodium hypophosphite (AR)	NaH_2_PO_2_·H_2_O	Aladdin, China
4	Stannous chloride (AR) Ascorbic acid (AR)	SnCl_2_·2H_2_O	Aladdin, China
5		C_6_H_5_O_6_	Aladdin, China
6	Hydroquinone (AR)	C_6_H_4_(OH)_2_	Aladdin, China
7	Hydrochloric acid (AR)	HCl	Aladdin, China
8	Pine Oil Alcohol (AR)	C_10_H_18_O	Aladdin, China
9	Ethyl cellulose (AR)	(C_12_H_22_O_5_)_n_	Aladdin, China
10	Sulfosalicylic acid (AR)	C_7_H_6_O_6_S·2H_2_O	Aladdin, China
11	Anhydrous ethanol (AR)	C_2_H_6_O	Aladdin, China
12	Span™ 85 (AR)	C_60_H_108_O_8_	Aladdin, China
13	Dibutyl phthalate (AR)	C_16_H_22_O_4_	Aladdin, China
14	Deionized water (AR)	H_2_O	Watsons, China
15	Polyethylene glycol (AR)	HO(CH_2_CH_2_O) H	Aladdin, China
16	Micron copper powder (3.0–4.5 μm)	Cu	Tianjiu, China
17	Polyvinyl pyrrolidone (AR)	(C_6_H_9_NO)_n_	Aladdin, China
18	Silver nitrate (AR)	AgNO_3_	Aladdin, China
19	Ferrous sulfate (AR)	FeSO_4_	Aladdin, China

## Data Availability

Not applicable.
